# The genetic basis for the selection of dairy goats with enhanced resistance to gastrointestinal nematodes

**DOI:** 10.1051/parasite/2017033

**Published:** 2017-08-09

**Authors:** Felix Heckendorn, Anna Bieber, Steffen Werne, Anastasios Saratsis, Veronika Maurer, Chris Stricker

**Affiliations:** 1 Research Institute for Organic Agriculture (FiBL) PO Box CH-5070 Frick Switzerland; 2 Laboratory of Parasitology, Veterinary Research Institute, Hellenic Agricultural Organization Demeter, Thermi 57001 Thessaloniki Greece; 3 agn Genetics GmbH Börtjistrasse 8b 7260 Davos Switzerland

**Keywords:** Dairy goat, Gastrointestinal nematode, Heritability, Genetic correlation, Phenotypic correlation, Production

## Abstract

Gastrointestinal nematodes (GIN) severely affect small ruminant production worldwide. Increasing problems of anthelmintic resistance have given strong impetus to the search for alternative strategies to control GIN. Selection of animals with an enhanced resistance to GIN has been shown to be successful in sheep. In goats, the corresponding information is comparatively poor. Therefore, the present study was designed to provide reliable data on heritabilities of and genetic correlations between phenotypic traits linked to GIN and milk yield in two major dairy goat breeds (Alpine and Saanen). In all, 20 herds totalling 1303 goats were enrolled in the study. All herds had (*i*) a history of gastrointestinal nematode infection, (*ii*) uniform GIN exposure on pasture and (*iii*) regular milk recordings. For all goats, individual recordings of faecal egg counts (FEC), FAMACHA^©^ eye score, packed cell volume (PCV) and milk yield were performed twice a year with an anthelmintic treatment in between. The collected phenotypic data were multivariately modelled using animal as a random effect with its covariance structure inferred from the pedigree, enabling estimation of the heritabilities of the respective traits and the genetic correlation between them. The heritabilities of FEC, FAMACHA^©^ and PCV were 0.07, 0.22 and 0.22, respectively. The genetic correlation between FEC and FAMACHA^©^ was close to zero and −0.41 between FEC and PCV. The phenotypic correlation between FEC and milk yield was close to zero, whereas the genetic correlation was 0.49. Our data suggest low heritability of FEC in Saanen and Alpine goats and an unfavourable genetic correlation of FEC with milk yield.

## Abstract (fr)

Les nématodes gastrointestinaux (NGI) ont un impact important sur la production des petits ruminants dans le monde entier. L’augmentation croissante des problèmes de résistance aux anthelminthiques a donné une forte impulsion à la recherche de stratégies alternatives pour contrôler les NGI. La sélection d’animaux ayant une meilleure résistance aux NGI s’est avérée efficace chez les brebis. Chez les chèvres, moins d’informations sont disponibles. Cette étude a donc été conçue pour fournir des données sur l’héritabilité et les corrélations génétiques entre les caractères phénotypiques liés aux NGI et à la production de lait chez deux races de chèvres laitières (Alpine et Saanen). Vingt troupeaux, totalisant 1303 chèvres ont été inclus dans l’étude. Tous les troupeaux avaient (i) des antécédents d’infestation aux nématodes gastrointestinaux, (ii) une exposition uniforme aux NGI sur pâture et (iii) des enregistrements réguliers de production laitière. Pour toutes les chèvres, des enregistrements individuels de nombres d’œufs fécaux (FEC), de FAMACHA et d’hématocrite ont été effectués deux fois par an avec un traitement anthelminthique intermédiaire. En incluant le pedigree des animaux, les données phénotypiques rassemblées ont été utilisées pour modéliser les héritabilités des traits respectifs et la corrélation génétique entre eux. Les héritabilités de FEC, FAMACHA et hématocrite étaient respectivement de 0.07, 0.22 et 0.22. La corrélation génétique entre FEC et FAMACHA était proche de zéro et de −0.41 entre FEC et hématocrite. La corrélation phénotypique entre la FEC et la production laitière était proche de zéro, tandis que la corrélation génétique était de 0.49. Nos données suggèrent une faible héritabilité de la FEC chez les chèvres Saanen et Alpine et une corrélation génétique défavorable entre FEC et production laitière.

## Introduction

Gastrointestinal parasitism is one of the most important diseases of ruminant livestock, affecting pasture-based production systems worldwide [38]. For decades, the control of gastrointestinal nematodes (GIN) relied essentially on the repeated use of anthelmintics. Amongst other factors, this practice has led to a rapid evolution of anthelmintic resistance in goats [40, 44, 54]) and sheep [25, 46]. The scientific community today largely agrees that the control of GIN depending solely on anthelmintics is not sustainable [30, 42]. This situation was and still is a strong driver of the search for more sustainable strategies to control GIN, either by reducing the use of anthelmintics to slow down the process of resistance development, or by trying to replace them completely.

Amongst the different strategies having emerged from these scientific efforts, the selection of genetically GIN-resistant hosts is particularly interesting, as it represents a permanent solution requiring no additional resources for maintenance. Genetic resistance is conferred by various immune effectors of the host which impact GIN within the host and limit their action [2]. The aim of selection for genetic GIN resistance is therefore to increase favourable alleles at genetic loci that are related to immune response. Already in the 1970s, it became clear that sheep could be selected for GIN resistance using faecal egg counts (FEC) as a phenotypic trait, because FEC proved to be a highly variable trait and also a good estimator of worm burden in small ruminants [24]. A number of long-term selection studies were initiated through the 1980s and 1990s, and have further substantiated the potential of FEC as a phenotypic marker for selection of GIN resistance [15, 52]. Subsequent work evaluated additional potential markers for GIN resistance such as faecal scores or dag scores [39]. In situations where *Haemonchus* spp. are the dominant GIN species, packed cell volume (PCV) and FAMACHA^©^ eye scores have been shown to be good selection traits [4, 32, 51].

Depending on the study, heritabilities for FEC in sheep have been reported to range between 0.08 and 0.43. For faecal score, dag score, FAMACHA^©^ and PCV, heritabilities of 0.12, 0.11, 0.55 and 0.29 have been estimated, respectively [13, 14, 35]. Proper measurement of these traits usually requires abstaining from anthelmintic treatments before recording. As a result, additional phenotypic traits for GIN resistance have recently been investigated in sheep. For example in 2012, Shaw et al. [47] identified salivary IgA reacting with a carbohydrate larval surface antigen (CarLA) as a suitable measure of protective immunity with a heritability of 0.3. In addition, a DNA marker linked to enhanced GIN resistance was marketed by Pfizer Animal Genetics, New Zealand (WormSTAR – see also [20]). Most studies investigating the genetics of GIN resistance in sheep also evaluated the genetic correlation with other economically important traits, particularly live weight gain. Although some authors have reported strong, negative (favourable) correlations between live weight gain and GIN resistance [3, 5], others have also noted positive (unfavourable) correlations between them [33, 34]. Generally, however, the correlation has been shown to be close to zero [23]. Overall, research efforts for the selection of GIN-resistant phenotypes in sheep have been substantial and today in Australia and New Zealand GIN-resistant sheep are successfully introduced into routine farming conditions [25].

Given the extensive knowledge that has emerged from sheep selection studies, it is surprising that information on the potential for selecting GIN-resistant goats is very limited. This may partly originate from early findings reported by Woolaston et al. [53] which showed that heritabilities of GIN resistance, as measured by FEC in Fijian goats, were low (i.e. 0.07). A number of studies have, however, been published in the last couple of years that seem to revise the early findings of Woolaston et al. [53]. Work from New Zealand with Saanen goats reported a direct heritability of 0.09 for FEC in mid-lactation and across years [28, 36], Mandonnet et al. [29] estimated heritabilities for FEC of 0.14 and 0.33 for 4-month- and 10-month-old Creole goats, respectively, and a Scottish study estimated the heritability of Cashmere goats to be 0.17 for a single FEC and 0.33 for the mean of five FEC measurements [49]. More recently, Mandal and Sharma [27] published a heritability of 0.13 for averaged repeated FEC in Barbari goats. Overall, these studies show that FEC heritabilities of goats seem comparable to those of sheep. However, reports remain rare and heritability estimates are sometimes based on few animals, which introduce a substantial degree of uncertainty. As far as the genetic correlations between FEC and production traits are concerned, knowledge is even more restricted. Vagenas et al. [49] found a slightly positive (unfavourable) correlation between FEC and different production traits (all linked to fibre) in Cashmere goats. Research from New Zealand in Saanen goats found no significant genetic correlations between milk traits (i.e. yield, content) and FEC, while phenotypic correlations between them were negative and small [36]. These findings contrast with results from France, where the phenotypic correlation between FEC and milk yield was positive [17]. Because the overall information on the genetic potential of goats to resist GIN is poor, the aim of the present study was to further explore the genetic basis of GIN resistance in two economically important dairy goat breeds (Saanen and Alpine) in Switzerland, by (*i*) producing reliable data on heritabilities of FEC, PCV and FAMACHA^©^, and by (*ii*) assessing the genetic correlation between these traits and milk production in a large-scale, multicentre field trial.

## Materials and methods

### Selection of study flocks

The study was performed over a two-year period in 2011 and 2012 and concerned goats carrying natural GIN infections on commercial farms. The first study year served as a pre-trial period with the objective of selecting a sufficient number of goat herds meeting the following criteria, to enter collected data in the second year: (*i*) only dairy goat herds of either the pure Alpine or pure Saanen breeds were eligible for the study, (*ii*) the herds needed to have a history of GIN-related disorders, (*iii*) daily feed intake on pasture had to be at least 50% such that the animals were sufficiently exposed to GIN, (*iv*) exposure to GIN needed to be uniform for every herd (i.e. common pasture), (*v*) milking performance data needed to be available for all female goats enrolled in the study and (*vi*) the average genetic relationship between animals in different flocks was maximised in order to increase statistical power to estimate genetic (co)variance components. Including related animals in the study is a prerequisite for estimation of genetic (co)variances. Due to the small herds present in Switzerland and the widespread use of natural service sires, this strategy was chosen to avoid the inclusion of genetically independent herds.

A first set of 35 herds meeting the parasitological inclusion criteria (*i*)*–*(*v*) was pre-selected from data derived from the parasite monitoring programme run by the Swiss Health and Extension Service for Small Ruminants. Pedigree data enabling the calculation of the average genetic relationship between the pre-selected flocks was provided by the Swiss Goat Breeding Association and reduced the initial set of 35 to 27 flocks. These flocks were monitored on two occasions in year 1 (early summer and autumn) in order to collect faecal material from 50% of all goats in each flock for up-to-date parasitological information (see Laboratory analysis section). Based on the obtained results, 20 flocks were finally selected as a basis for full data collection in year 2.

### Study design and phenotyping

The 20 goat flocks selected were visited by trained personnel for individual phenotyping of all goats > 10 months of age. The first visit in year 2 took place in early summer, 3.5–4.5 months after turnout on pasture. Blood was collected by jugular vein puncture of each goat for subsequent PCV determination. FAMACHA^©^ eye scores were individually recorded as described by Van Wyk and Bath [50] and by using a headlight, assuring homogeneous light conditions while scoring. Furthermore, faecal samples were individually drawn from the rectum of each goat for parasitological analysis (see below). Finally, the live weight of all goats was recorded. After this first sample/data collection, all study animals in all flocks were dewormed, irrespective of their FEC. This procedure was chosen so that the second sampling/data collection in early autumn (3.5–4.5 months after anthelmintic treatment) would be performed on a GIN population independent of the one present at first sampling/data collection. Anthelmintic treatment was administered by trained personnel either as an oral levamisole/triclabendazole drench (Endex^®^, Novartis Animal Health, Switzerland) at a dose of 11.25 mg levamisole hydrochloride/kg live weight or with topically applied eprinomectin (Eprinex^®^, Merial, France) at a dose of 1 mg eprinomectin/kg live weight [8]. The respective goat farmers made the choice of the anthelmintic used. Because of the unclear anthelmintic resistance status of the study herds, a faecal egg count reduction test (FECRT) was performed on approximately 20 goats with FEC > 300 in each flock. FECRT and bootstrap confidence intervals were calculated using the “eggCounts” R-package [48], according to the World Association for the Advancement of Veterinary Parasitology (W.A.A.V.P.) guidelines [9, 10]. This information was subsequently used to model the effect of incomplete clearing of GIN infection by anthelmintic treatment.

At the farm visit in autumn, with the exception of live weight recording, the same set of sample/data as in early summer was collected for every goat.

All animal-related procedures were in compliance with the Swiss animal welfare act and the animal welfare ordinance, as well as the animal experimentation ordinance, and these procedures were approved by the responsible authority (Cantonal Veterinary Office, Aargau, Switzerland; permission No. 75’644).

### Animal-related data

The Swiss Goat Breeding Association provided performance and pedigree data for all animals enrolled in the study for the whole year of full data collection (2012) in order to calculate genetic correlations between production and auxiliary traits for GIN resistance. Data included birth date, litter date, breed, lactation number (primiparous or multiparous), lactation milk yield for the year 2012 (standardised to 220 days), as well as the test day milk yield measured on the day closest to each date of phenotyping. The breed was either Saanen or Alpine, or set to missing otherwise. Data were filtered for consistency by considering lactation milk yield only up to a maximum of 1500 kg and fat and protein percentage to a maximum of 6% each. Values exceeding these thresholds were set to missing in order to minimise the inclusion of inconsistent outliers (data errors). Lactation milk yield and test day milk yield were considered only for animals with a nematode-related phenotype on FEC, FAMACHA^©^ or PCV. Furthermore, with respect to data on test day milk yield, only the observation closest to the time of nematode scoring was used. The full pedigree for all phenotyped animals comprised a total of 10,039 animals: 1488 founder animals were of the Saanen breed, 1925 were Alpine goats. Among the animals scored either for FEC, FAMACHA or PCV, there were 353 Saanen and 950 Alpine goats.

### Laboratory analysis

Individual faecal samples collected in the pre-trial period (i.e. early summer and autumn of year 1) were analysed for GIN using a McMaster procedure as described by Schmidt [45]. Samples were separately bulked (bulks of five samples) for primiparous and multiparous goats. Additionally, for each flock 50% of individual faecal samples were bulked (2 g of fresh faeces per individual sample) for faecal culture and subsequent differentiation of *Haemonchus contortus* and remaining GIN genera [26].

Faecal samples collected in year 2 were individually analysed for GIN eggs using the quantitative McMaster technique described above. Faecal bulks were set up as in year 1 but GIN eggs were isolated from bulks and PNA (peanut agglutinin) stained for differentiation between *Haemonchus contortus* and other GIN eggs as described in [21]. PNA staining was also used to determine the percentage of *Haemonchus contortus* eggs in bulk faecal samples pre- and post anthelmintic treatment when performing the FECRT. PCV was determined from ethylenediaminetetraacetic acid (EDTA) blood using a Pico 17 Hematocrit Rotor (Thermo Fisher Scientific).

### Data management and statistical analysis

All phenotypic data were checked for consistency. For statistical analysis, observations for FEC, FAMACHA and PCV were discarded if FEC > 10,000 EpG (eggs per gram), FAMACHA^©^ score < 1 or > 5 and/or PCV < 14% or > 50%. This resulted in 19 observations for FEC, 25 for FAMACHA^©^ and 47 for PCV being set to missing. The thresholds were chosen in order to minimise the inclusion of outliers. For all statistical analyses, FEC was transformed to be approximately normally distributed using the transformation FECtrans = (FEC + 1)^0.36^. For FEC related to *Haemonchus contortus* only, FEC_haem was obtained as FEC × Prop_H.contortus, where Prop_H.contortus was the proportion of *Haemonchus contortus* as determined by [21]. FECtrans_haem was then obtained as (FEC_haem + 1)^0.36^. First, FECtrans and FECtrans_haem, FAMACHA^©^ and PCV were fitted as repeated measures in a multivariate linear model using breed, season (summer, autumn), type of anthelmintic (levamisole hydrochloride/triclabendazole or eprinomectin), FECRT (six classes; proportion of FEC reduction after treatment being < 20%, < 40%, < 60%, < 80%, ≤ 100%) and birth date (three classes; born before October 2003, born between October 2003 and September 2005, born after September 2005) as fixed effects. Animal, permanent environment (to account for repeated measurements taken on the same animal), herd and classifier (for FAMACHA^©^ scores only) were considered as random effects. This model is referred to as MV3. For the second model, MV4L, model MV3 was expanded by a fourth dependent variable, lactation milk yield for the lactation during which the GIN-related traits were measured. Lactation number was used as an additional fixed effect and herd as an additional random effect in MV4L. The third and last model (MV4TD) was similar to the second model, but used milk yield at the test day closest to recording of GIN-related phenotypes instead of lactation milk yield as an additional variable. As GIN-related phenotypes were measured twice on each animal, different test day milk yields corresponded to those GIN-related phenotypes. Therefore, test day milk yields were considered as repeated observations of the same trait and thus, a random permanent environmental effect for test day milk yield was introduced in MV4TD. MV4TD was the only model used for the trait FECtrans_heam. The effects considered in the models were assumed to be normally distributed with expected values,

Eap1p2h1h2e=000000 and CoVariances Varap1p2h1h2e=G✪A000P1✪I000σp22✪I00H1σI000σH22✪I000R✪I

where *G* is the additive genetic covariance matrix between the traits, A is the additive genetic relationship matrix, *P*1 is the covariance matrix for the permanent environment related to FEC, FAMACHA^©^ and PCV, *σ* ^2^_p2_ is the variance of the permanent environment for test day milk yield, *H*1 is the covariance matrix for the herd related to FEC, FAMACHA^©^ and PCV, *σ* ^2^_H2_ is the variance of the herd related to test day or lactation milk yield, *R* is the residual covariance matrix between all four traits and ✪ denotes the Kronecker product. All analyses were performed using the remlf90 and aireml90 software package [31]. Estimates of effects and standard errors were computed using aireml90, whereas variance components were estimated using remlf90.

## Results

### Phenotypic data

**Figure 1.**
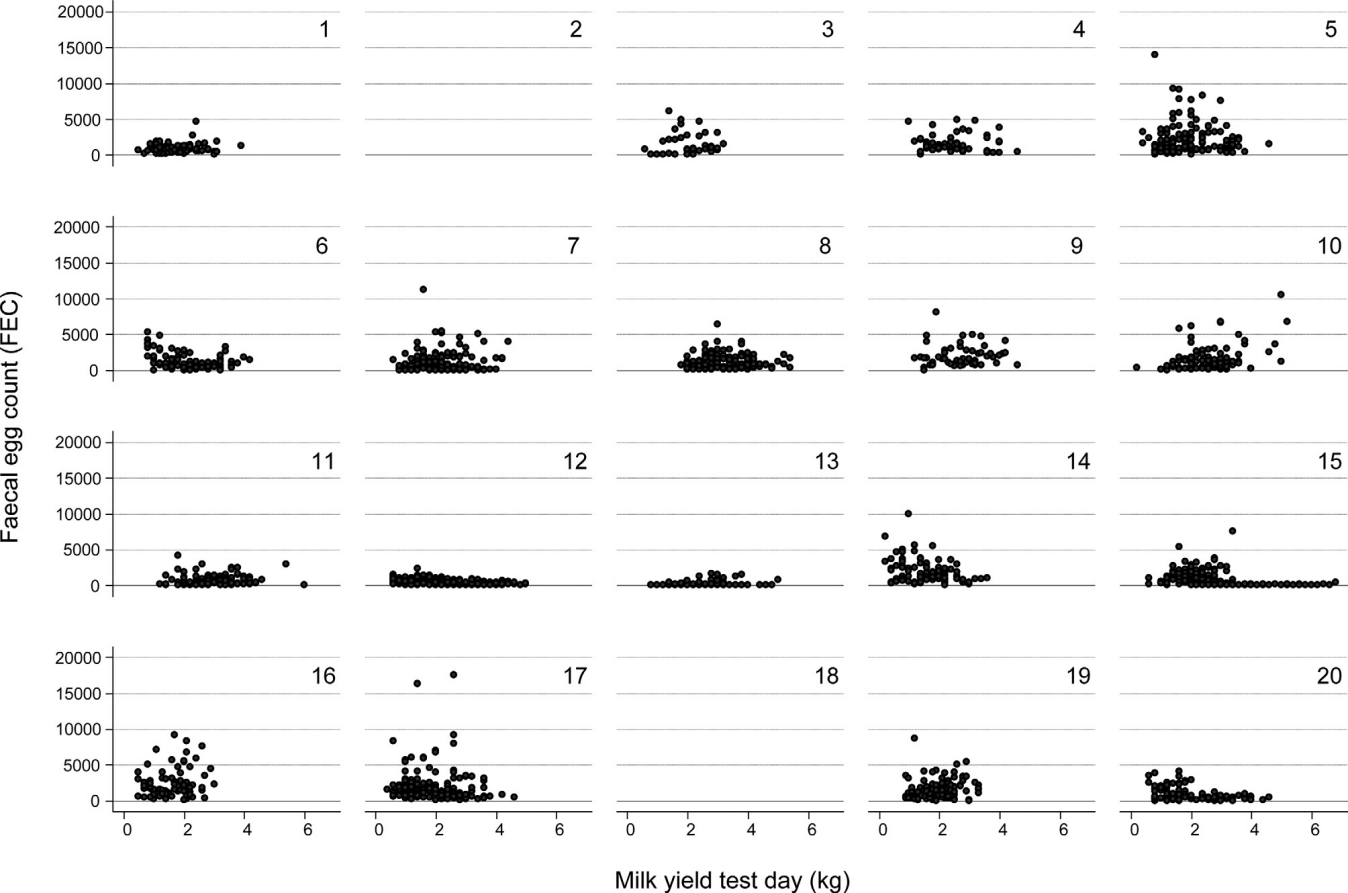
Scatterplots of phenotypic measures for test day milk yield versus FEC (as measured by eggs per gram [EpG] of faeces) for all goat herds enrolled in the study. Note that for herd numbers 2 and 18, no performance data were available, which is due to the decision of the farmer to discontinue milk performance recording after initiation of the study. Note further that outliers for FEC were plotted.

**Figure 2.**
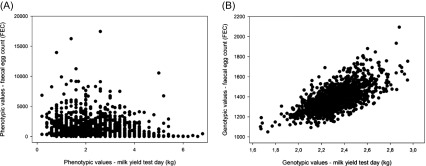
Phenotypic (A) and genotypic (B) values for faecal egg count (back-transformed FEC) as measured by eggs per gram (EpG) of faeces and milk yield test day (kg). Note that for the plot of phenotypic values, FEC outliers were also plotted.

FEC, FAMACHA^©^ eye scores and PCV were collected/determined for 1303 animals from 20 herds. One hundred and fifty-five animals were phenotyped only once and 1145 animals had two phenotypic recordings. Detailed information on FEC, FAMACHA^©^ and PCV across herds and for both sampling dates is given in Table 1. For 15 herds, FECtrans differed significantly between the first and the second sampling (early summer and autumn). For the two sampling periods, the mean *Haemonchus contortus* percentage within the GIN population was 53% (early summer) and 56% (autumn), and ranged between 4% and 86% and 4% and 93% depending on the study herd (see Table 1). Milk production data were available for 1221 goats (912 Alpine, 309 Saanen) from 18 herds due to the decision of two farmers to discontinue milk performance recording after initiation of the study; lactation milk yield (220 days) ranged between 185 and 1346 kg milk for the entire lactation, with an across-herd mean of 649 ± 190 kg milk. Milk yield differed significantly between herds (range of means: 446–822 kg). Milk yield on the test day closest to GIN phenotyping in relation to FEC is presented in Figure 1. There was no significant phenotypic correlation between FEC and test day milk yield, whether for individual herds or for pooled overall data (Fig. 2).

**Table 1.** Mean faecal egg counts (FEC), FAMACHA scores (FAM, score ranging from 1 [red conjunctiva] to 5 [white conjunctiva]) and packed cell volumes (PCV) determined on all goats in 20 herds in early summer and autumn. ALP = Alpine, SAA = Saanen, *SD* = standard deviation, na = not analysed. *Haemonchus contortus* percentage (H.c.%) was determined on a herd basis. Deviations in animal numbers between the early summer and autumn sampling are due to deaths and/or acquisition/selling of animals.

			Early summer					Autumn			
Herd	Goats (*n*)	Breed	FEC ± *SD*	H.c.%	FAM ± *SD*	PCV ± *SD*	Goats (*n*)	FEC ± *SD*	H.c.%	FAM ± *SD*	PCV ± *SD*
1	45	ALP/SAA	1160 ± 740	26	3.5 ± 0.6	31.1 ± 3.2	48	660 ± 390	20	3.9 ± 0.6	31.3 ± 2.6
2	44	ALP	1270 ± 970	48	3.2 ± 0.5	28.3 ± 2.8	44	1310 ± 1040	17	3.8 ± 0.6	23.8 ± 4.0
3	34	SAA	1400 ± 900	74	3.1 ± 0.6	27.4 ± 2.9	34	1530 ± 1920	92	3.0 ± 0.8	29.0 ± 4.0
4	29	ALP	1530 ± 1420	56	3.3 ± 0.7	30.8 ± 3.1	29	1430 ± 1040	73	2.9 ± 0.7	31.8 ± 6.5
5	63	SAA	2250 ± 2420	61	3.5 ± 0.6	27.3 ± 6.0	61	2280 ± 1970	35	3.2 ± 0.6	28.1 ± 4.2
6	47	ALP	900 ± 940	18	2.6 ± 0.6	27.6 ± 4.9	41	1840 ± 1310	19	3.1 ± 0.6	30.2 ± 2.8
7	90	ALP	170 ± 330	40	2.9 ± 0.5	30.6 ± 4.9	94	1610 ± 1650	74	3.9 ± 0.5	28.3 ± 6.4
8	90	ALP	710 ± 730	50	2.9 ± 0.7	27.0 ± 6.0	85	1230 ± 1060	72	3.5 ± 0.5	25.1 ± 3.2
9	28	SAA	2740 ± 1700	64	3.1 ± 0.6	30.4 ± 3.1	28	1270 ± 720	60	3.7 ± 0.6	27.3 ± 2.6
10	48	ALP/SAA	1990 ± 2180	84	2.8 ± 0.6	28.1 ± 3.8	50	1150 ± 1330	90	3.4 ± 0.7	29.7 ± 3.0
11	67	ALP	680 ± 630	86	2.9 ± 0.6	28.6 ± 3.8	63	600 ± 750	88	3.7 ± 0.7	28.6 ± 3.2
12	136	ALP	280 ± 220	6	2.8 ± 0.6	29.6 ± 3.2	144	580 ± 360	14	3.4 ± 0.6	31.3 ± 3.0
13	48	ALP/SAA	410 ± 810	64	3.0 ± 0.3	29.1 ± 4.6	46	50 ± 110	na	3.1 ± 0.8	27.0 ± 2.3
14	54	ALP	1920 ± 1740	4	3.2 ± 0.5	26.6 ± 5.0	48	2220 ± 2930	4	3.9 ± 0.5	27.8 ± 3.1
15	96	ALP	230 ± 870	48	2.9 ± 0.4	30.7 ± 4.4	89	1200 ± 960	69	3.2 ± 0.6	27.7 ± 3.6
16	43	ALP	2830 ± 2290	70	3.4 ± 0.7	25.7 ± 4.4	39	1970 ± 1440	23	3.5 ± 0.7	24.6 ± 3.0
17	74	ALP	2260 ± 3070	72	3.1 ± 0.6	26.8 ± 4.2	72	1860 ± 1380	57	3.6 ± 0.6	28.1 ± 3.7
18	75	SAA	3950 ± 3150	58	3.0 ± 0.3	27.7 ± 3.9	83	1530 ± 1240	56	3.2 ± 0.7	30.0 ± 3.0
19	59	ALP/SAA	1710 ± 1300	69	2.9 ± 0.6	25.8 ± 4.3	60	1150 ± 1320	62	3.3 ± 0.6	27.8 ± 4.2
20	58	SAA	370 ± 270	8	3.4 ± 0.5	30.8 ± 3.2	56	1260 ± 1090	93	3.4 ± 0.6	27.3 ± 3.2

### Faecal egg count reduction

Faecal egg count reduction (FECR) results are presented in Table 2. Out of six FECRT with levamisole hydrochloride (Endex^®^), five FECRT revealed effectiveness of the drug close to 100%. The anthelmintic efficacy was much more variable for eprinomectin (Eprinex^®^), with a mean FECR of 55% for the 14 tests performed with this anthelmintic. In cases where PNA staining and the determination of *Haemonchus contortus* eggs were possible post-treatment, in virtually every case an increase in *Haemonchus contortus* percentage was seen when compared to the pre-treatment counts.

**Table 2.** Number of goats enrolled in faecal egg count reduction tests (FECRT) within the 20 experimental farms. EPR = eprinomectin, LEV = levamisole, FEC = faecal egg count, pre-t = pre-treatment, post-t = post-treatment. *Haemonchus contortus* was determined in bulk faecal samples of animals enrolled in the FECRT of each farm and determined with PNA staining. For the sake of clarity, FECRT with levamisole and eprinomectin were grouped. As a consequence, herd numbers do not correspond to those in Table 1.

Herd	Goats (*n*) FECRT	Anthelmintic	FEC pre-t (min-max)	FEC post-t (min-max)	FECR %	(95% CI)	H.c.% pre-t	H.c.% post-t
1	20	EPR	1780 (1300–4700)	393 (0–1100)	78	(69, 85)	26	86
2	20	EPR	2225 (1250–5200)	855 (0–1100)	62	(51, 71)	48	83
3	19	EPR	4608 (2250–13,950)	784 (0–2100)	83	(75, 89)	61	91
4	16	EPR	1609 (950–3300)	34 (0–250)	98	(95, 100)	18	nn
5	17	EPR	712 (300–1700)	398 (50–650)	44	(26, 58)	40	83
6	20	EPR	2002 (1150–4150)	2682 (0–4950)	–34	(−63, 9)	50	97
7	20	EPR	3960 (1150–10,550)	4723 (1950–13,050)	–19	(−48, 6)	84	95
8	20	EPR	1445 (950–2400)	960 (0–3000)	34	(7, 37)	86	96
9	17	EPR	1144 (350–4950)	914 (0–2800)	8	(–18, –44)	64	90
10	20	EPR	3588 (1900–9900)	421 (0–3050)	88	(79, 95)	4	2
11	20	EPR	1030 (500–7500)	73 (0–600)	93	(84, 97)	48	nn
12	20	EPR	4928 (2550–9150)	2650 (0–6150)	46	(27, 65)	70	85
13	17	EPR	7124 (5100–12,350)	4763 (350–13,250)	42	(27, 54)	58	86
14	20	EPR	715 (500–1350)	73 (0–350)	90	(85, 95)	8	92
15	20	LEV	2208 (1250–3050)	13 (0–100)	99	(99, 100)	74	nn
16	20	LEV	2143 (550–4850)	115 (0–350)	95	(91, 97)	56	nn
17	20	LEV	3503 (1800–8050)	3 (0–50)	100	(–, –)	64	nn
18	20	LEV	715 (550–1100)	25 (0–150)	97	(94, 99)	6	nn
19	20	LEV	3483 (1450–9100)	23 (0–150)	99	(99, 100)	72	nn
20	20	LEV	2433 (1300–4050)	15 (0–150)	99	(99, 100)	69	nn

### Parameter estimates

Heritabilities, phenotypic and genetic correlations between FECtrans, FAMACHA^©^, PCV and milk yield for models MV3, MV4L and MV4TD are presented in Table 3. Heritabilities for FECtrans_haem decreased slightly and the genetic correlations between the pathophysiological traits (i.e. FAMACHA^©^, PCV) and FEC were more pronounced (Table 2). Also, the genetic correlation between test day milk yield and FECtrans_haem was stronger (*r*_g_ = 0.63) compared to the genetic relation of overall FECtrans with production (*r*_g_ = 0.49). Phenotypic correlations between FECtrans/Fectrans_haem and milk yield were null or slightly negative, while the genetic correlation was positive. The genetic relation between FEC and test day milk yield is shown in Figure 2. Additional AIREML estimates for effects in models MV3, MV4L, MV4TD and MV4TD for FECtrans_haem are given in supplementary files available at http://www.parasite.org/10.1051/parasite/2017033/olm.

**Table 3.** Heritabilities (on diagonal, bold, dark grey), genetic correlations (above diagonal, grey) and phenotypic correlations (below diagonal, light grey) for the traits FECtrans, FAMACHA, PCV and milk yield for models MV3, MV4L and MV4TD in the upper section and for the traits FECtrans_haem, FAMACHA, PCV and test day milk yield for model MV4TD in the lower section. In each cell, the upper line is the parameter estimate, the lower line is its standard error. “*” indicates that the parameter estimate is significantly different from zero on the 5% level using a *t-*test. Note that phenotypic correlations are not model-dependent, thus the cells in the lower triangle of the table are merged across models with a single estimate per cell.

	FECtrans			FAMACHA			PCV			Milk yield		
	MV3	MV4L	MV4TD	MV3	MV4L	MV4TD	MV3	MV4L	MV4TD	MV3	MV4L	MV4TD
FECtrans	.07*	.07*	.07*	−.03*	−.02	−0.01	−.39*	−.40*	−.41*	–	.19	.49*
	***.01***	.***03***	***.01***	.01	*.23*	.01	.01	*.20*	.01		.*17*	.01
FAMACHA	0.18*			**.22**	.22*	0.22*	−.60*	−.60*	−.60*	–	.17	.11*
	*.02*			***.42***	**.*04***	**.*01***	.01	.*11*	.01		.*10*	.01
PCV	−.27*			−.17*			.22*	.22*	.22*	–	−.39*	−.35*
	*.02*			*.02*			***.01***	***.04***	***.01***		.*1*	.01
Milk yield	.01			.22*			−.07*			**–**	.28*	.12*
	*.02*			*.02*			*.02*				***.07***	***.02***
	FECtrans_haem			FAMACHA			PCV			Milk yield TD		
FECtrans_haem			**.04**			.22*			−.55*			.63
			***.03***			*.01*			*.01*			*.01*
FAMACHA	.16*					.20*			−.61*			.12
	*.02*					***.00***			*.01*			*.01*
PCV	−.29*			−.17*					.22*			−.28
	*.02*			*.02*					***.01***			*.01*
Milk yield	−.10*			−.08*			−.09*					.12*
	*.02*			*.02*			*.02*					***.00***

## Discussion

The main finding of this study is that FEC and test day milk yield are genetically correlated in an unfavourable way (*r*_g_ = 0.49). This contradicts the findings of an early study by Morris and Wheeler [36] in Saanen dairy goats with a comparable, pasture-based design, where genetic correlations were neutral or even slightly negative (i.e. favourable, *r*_g_ = −0.21). The main difference between our study and the work of Morris and Wheeler [36] relates to the GIN species present in study animals. *H. contortus* was most prevalent (around 50% of total FEC) in our trial, whereas *Trichostrongylus* spp. and *Teladorsagia* spp. (together > 74%) were the main GIN species in Morris’s study. This could partly explain the pronounced positive correlation found in our study and is further substantiated by the fact that the genetic correlation between FEC specific to *H. contortus* and test day milk yield in our study was stronger (i.e. *r*_g_ = 0.63) than for overall FEC. Another possibility to explain the discrepancy in results between our study and the work by Morris et al. relates to potential differences in pasture infection pressure with GIN. In a modelling study, Doeschl-Wilson et al. [12] showed in sheep that the genetic correlation between GIN FEC and production changes from favourable to unfavourable with increasing infection pressure. Although it is not clear whether the same applies to dairy goats, this could be a starting point to explain the observed difference in genetic correlation between the two studies. To study such genotype-by-environment (G × E) interactions, observations from environments with high and low infection pressures should be obtained. Then, a simple strategy of investigating G × E interactions would be to model GIN resistance and performance traits jointly in these two environments as separate, correlated traits. A genetic correlation deviating from 1 would then indicate G × E interactions for GIN resistance, performance traits or both. However, in the present study, observations were from routine Swiss production environments lacking sufficient observations from low-infection pressure environments as indicated by Figure 1 for the 20 herds sampled. Therefore, targeting whether there is significant G × E interaction actually changing the genetic correlation between resistance and production traits is not feasible with the present data. This type of study performed with real data would of course be very valuable, as Doeschl-Wilson [12] have shown the above-mentioned effect of G × E interaction *in silico*.

The comparison of genetic correlations between phenotypic traits related to GIN parasitism and production traits other than milk is not straightforward. This is primarily because we are dealing with other breeds and possibly also differences in underlying immunological responses. On the other hand, some studies were, as in our study, carried out in environments with moderate to high GIN pressure on pasture and are therefore worthy of mention. In fibre goats, Vagenas et al. [49] reported positive (i.e. unfavourable) genetic correlations between FEC and fleece parameters ranging between 0.16 and 0.30, depending on the fleece trait. Also for meat producing East African goats, the correlation between FEC and live weight gain was 0.25 for 12-month-old animals [1]. Taken together, the available information suggests that in situations where goat production involves pasturing and medium to high GIN infection pressure, the selection of genetically superior goats for production (in our case milk yield) will result in a correlated undesired selection response for GIN susceptibility. To overcome this expected negative selection response for GIN susceptibility when goats are selected for performance, it is mandatory to evaluate the breeding population genetically for FEC and to select animals with a favourable genotypic value for both GIN resistance and performance.

Although there is an unfavourable genetic correlation between FEC and milk yield in our study, the phenotypic correlation between these traits is slightly negative, thus masking the unfavourable genetic relationship (see Table 3, Fig. 2). This points to negative environmental covariances between FEC and milk yield, i.e. environments with higher infection pressure leading to lower milk yield and vice versa (e.g. sub-optimal pasture management leading to high infection pressure and low quality feed caused by such sub-optimal management leading to low milk yield). Genetically susceptible animals, however, tend to be genetically superior with respect to milk yield. These opposite correlations (environmental and genetic) may result in the observed phenotypic correlation being close to zero. The neutral phenotypic correlation between FEC and milk yield also contradicts a series of French field studies with Alpine goats, which clearly showed that high producing goats have significantly higher FEC than low producers [7, 16–18]. There are, however, several differences between our study and the work performed in France. First, only the study by Hoste et al. [17] was based on natural GIN infections with goats in real-world production situations. Second, mean FEC of the 14 goat herds investigated by Hoste et al. [17] were considerably lower when compared to our study, which may have an influence on the immunity and the resilience pattern (see Hoste et al. [19] and Coop & Kyrizakis [11] for further information). Lastly, in the field study by Hoste et al. [17], the phenotypic correlation between all milk production and FEC data is not given, making a direct comparison impossible.

The design of our study included an anthelmintic treatment of all goats between the two phenotyping events. As we were unsure about the anthelmintic resistance status of the GIN populations in the 20 herds, we decided to check the efficacy of the two anthelmintics used within the trial (levamisole hydrochloride and epinomectin). Although the anthelmintic resistance status was not a core objective of the study, it is still worth mentioning that the mean FECR for eprinomectin-treated goats was as low as 55%. This is comparable with the findings of Murri et al. [37], who found a prevalence of anthelmintic resistance of 40% for the same drug. As discussed by Murri et al., the high prevalence of Eprinomectin resistance is related to the fact that this drug in Switzerland is registered for goats with a zero milk withdrawal period; with the consequence of highly frequent use by farmers in the past decade. We also showed that *Haemonchus contortus* percentages in the investigated GIN populations increased from the pre- to the post-treatment period indicating a higher prevalence of *Haemonchus contortus* resistance compared to the other GIN genera. Our study also confirmed that levamisole overall still has satisfactory efficacy in goats in Switzerland (compare with [37]).

Among the phenotypic traits investigated in our study, FEC is clearly the target trait to improve when selecting goats for enhanced genetic resistance to GIN. Compared to FAMACHA^©^ scores and PCV, which both reflect the pathophysiological consequences of GIN infection and particularly *Haemonchus* spp., FEC is directly linked to the actual GIN worm burden [6]. Compared to phenotyping FEC, PCV and even more so FAMACHA^©^ scores are less expensive traits to phenotype. Thus, PCV and FAMACHA^©^ were chosen in our study in order to evaluate their potential to replace FEC as phenotypes for selection in situations with moderate to high *Haemonchus* spp. prevalence. Unfortunately, they showed low to zero genetic correlations with FECtrans (around −0.41 and −0.01, respectively) and higher but still low genetic correlations with FECtrans_haem (−0.55 and 0.22). Therefore, at least in situations with medium proportions of *Haemonchus* spp., they are not suitable to serve as auxiliary traits to select for GIN resistance. Other authors have shown strong phenotypic correlations between both PCV and FEC, and FAMACHA^©^ and FEC in goats where the predominant GIN species was *Haemonchus* spp. [22, 44]. However, *Haemonchus* spp. proportions in the mentioned studies were clearly higher (between 75% and 95%) when compared to those found in our study (overall mean of approx. 50%), which might explain the rather weak relation between FAMACHA^©^/PCV and FEC in our work.

The genes of animals in the present study explained around 7% of the total phenotypic variance for FECtrans. This finding is in agreement with the study carried out by Morris and Wheeler [36] who found a heritability of 9% for FECtrans in Saanen goats. Mandonent et al. [29] found a comparable FEC heritability in Creole goats (i.e. 0.1). A heritability of around 10%, although low, still allows successful selection for GIN resistance. A strategy to select for a trait with low heritability is to use (offspring) means instead of individual observations. This strategy to increase the heritability of a trait is well known in animal breeding as progeny testing (e.g. Robertson and Rendel [41]). However, it requires large numbers of animals to be phenotyped, which limits its application to FEC. Another strategy to increase the heritability of a trait is to measure it under standardised conditions, eliminating the variance explained by environmental factors, such as herd, and trying to minimise the residual variance through standardised conditions. One could envisage male goats and their offspring being phenotyped at a young but immune-competent age under standardised conditions. Then, based on their genetic evaluation for GIN resistance, the best among them are distributed among the breeding flocks for further (progeny) testing. Based on the findings presented in this paper, increasing heritability by applying progeny testing under standardised conditions seems a promising approach to evaluate goats genetically for GIN resistance. The resulting accurate breeding values can then be used directly for selection. They may also serve as accurately measured (de-regressed) phenotypes for genome-wide association studies (e.g. Schaeffer [43]) using high-density single nucleotide polymorphisms to further identify quantitative trait loci underlying GIN resistance in goats in the future.

## Supplementary Material

AIREML estimates MV3 AIREML estimates MV4L AIREML estimates MV4TD AIREML estimates MV4TD for FECtrans_haem
