# Supplementary material for: The genetic basis for the selection of dairy goats with enhanced resistance to gastrointestinal nematodes
Source: Parasite. 2017 Aug 9;24:32. doi: 10.1051/parasite/2017033 (PMC5550108; doi:10.1051/parasite/2017033)
Supplement: Supplementary file 1 — AIREML estimates MV3 [file parasite-24-32-s1.pdf]

```
AIREML estimates for effects in model MV3
-------------------------------------------

Traits are 1 = FECtrans, 2 = FAMACHA, 3 = PCV

Random effects in model are
1 = herd where GIN phenotypes were scored
6 = classifier for FAMACHA-scores

Final estimate for covariance matrix for effect 1
   8.5008      0.29736      -1.7896
  0.29736      0.58627E-01 -0.29078E-01
  -1.7896     -0.29078E-01   2.0400

Final estimate for covariance matrix for effect 6
   0.0000       0.0000       0.0000
   0.0000      0.20936E-01   0.0000
   0.0000       0.0000       0.0000

Final estimate for residual covariance matrix
   18.135      0.43044      -5.7726
  0.43044      0.23022     -0.36370
  -5.7726     -0.36370       12.323

The solutions for the random effect 2 (animal) and 4 (permanent
environment) are not listed below, their covariance matrices are:

Final estimate for covariance matrix for effect 2
   1.8561     -0.19474E-01 -0.83368
 -0.19474E-01  0.91322E-01 -0.35622
 -0.83368     -0.35622       3.6564

Final estimate for covariance matrix for effect 4
  0.17771      0.14739E-01  0.30441
  0.14739E-01  0.12144E-02  0.25247E-01
  0.30441      0.25247E-01  0.52142


Fixed effects in model are
3  = season (1=spring, 2=fall)
5  = breed (1=Saanen, 2=Alpine)
7  = type of anthelmintic (1=Eprinex, 2=missing, 3=Endex)
8  = FECRT (1=1%-20% reduction after treatment, 2=missing,
3=96%-100%, 4=81%-95%, 5=21%-40%, 6=61%-80%, 7=41%-60%)
9 = birth date (age class 1=291-980 days, 2=older than 980 days,
3=1-290 days)

trait  effect    level     solution            s.e.
   1   1         1        -5.23978148          1.06279652
   2   1         1         0.05692273          0.11951962
   3   1         1         0.45485175          0.77701935
   1   1         2         0.61661489          0.87306925
   2   1         2        -0.13074164          0.09380916
   3   1         2         0.15164304          0.60655578
```

1	1	3	1.21071383	0.91756405
2	1	3	0.12118169	0.10271457
3	1	3	-2.31543818	0.64637176
1	1	4	2.17759376	0.97210178
2	1	4	0.35317991	0.11197297
3	1	4	-0.44004742	0.70857468
1	1	5	4.07706569	0.90666275
2	1	5	0.00615853	0.09536041
3	1	5	-0.16636251	0.61189958
1	1	6	3.27731779	1.10370350
2	1	6	0.28219370	0.13119952
3	1	6	-0.18029012	0.84121859
1	1	7	-3.56452584	0.82140159
2	1	7	-0.35638427	0.09213825
3	1	7	2.06899022	0.58837439
1	1	8	1.06442667	0.96531144
2	1	8	0.28036982	0.10922004
3	1	8	2.53634665	0.70862374
1	1	9	1.11316479	1.00369524
2	1	9	0.06146308	0.11276697
3	1	9	-0.83340690	0.72187333
1	1	10	0.41261762	0.99030400
2	1	10	-0.00162655	0.11235687
3	1	10	2.06808102	0.73004837
1	1	11	1.50170582	0.93729647
2	1	11	0.24974420	0.11407598
3	1	11	-0.81740326	0.73192647
1	1	12	-0.12488945	0.95432343
2	1	12	-0.46141359	0.11241391
3	1	12	-1.14859879	0.72298035
1	1	13	4.26109884	0.95030023
2	1	13	0.17862205	0.10621053
3	1	13	-1.33154329	0.68976656
1	1	14	-2.88313059	0.91447002
2	1	14	0.15313020	0.10654200
3	1	14	0.67365195	0.68842444
1	1	15	1.35021066	0.92502337
2	1	15	-0.02962118	0.10241890
3	1	15	-0.10291046	0.66260049
1	1	16	2.16669770	0.84199404
2	1	16	-0.07532718	0.09099572
3	1	16	-1.31184765	0.58723030
1	1	17	-0.90491616	0.85545827
2	1	17	-0.29600096	0.09308323
3	1	17	-1.41754327	0.60311694
1	1	18	-2.58307984	0.90704789
2	1	18	-0.14652676	0.10750307
3	1	18	0.30355693	0.68888884
1	1	19	-3.02411608	0.85183653
2	1	19	-0.01316326	0.09542017
3	1	19	0.48934275	0.60971181
1	1	20	-4.90478861	0.85030136
2	1	20	-0.23216052	0.09363325
3	1	20	1.31892752	0.60373554

1	3	1	7.67612179	1.30021349
2	3	1	2.79230908	0.20777189
3	3	1	27.70484820	0.99753416
1	3	2	7.97441579	1.35503055
2	3	2	3.13314927	0.17981283
3	3	2	28.39321414	1.04796644
1	5	1	-0.53357117	0.78883591
2	5	1	-0.38812759	0.10346035
3	5	1	0.90301074	0.67805907
1	5	2	0.00000000	0.00000000
2	5	2	0.00000000	0.00000000
3	5	2	0.00000000	0.00000000
1	6	1	0.00000000	0.00000000
2	6	1	0.00000000	0.14469316
3	6	1	0.00000000	0.00000000
1	6	2	0.00000000	0.00000000
2	6	2	0.15654400	0.08875966
3	6	2	0.00000000	0.00000000
1	6	3	0.00000000	0.00000000
2	6	3	-0.06206082	0.09287304
3	6	3	0.00000000	0.00000000
1	6	4	0.00000000	0.00000000
2	6	4	-0.09448317	0.09497062
3	6	4	0.00000000	0.00000000
1	7	1	0.86001737	0.73111613
2	7	1	0.27465158	0.08887007
3	7	1	-0.00496139	0.61490238
1	7	2	0.00000000	0.00000000
2	7	2	0.00000000	0.00000000
3	7	2	0.00000000	0.00000000
1	7	3	0.28005850	0.59223880
2	7	3	0.20491126	0.07223220
3	7	3	-2.62655663	0.51332678
1	8	1	-0.82523925	1.23733700
2	8	1	0.03784572	0.14458277
3	8	1	-1.84992255	1.01552829
1	8	2	3.45852834	1.07623507
2	8	2	0.33875062	0.12815309
3	8	2	0.54131263	0.87970467
1	8	3	3.83025505	1.06847131
2	8	3	0.20027876	0.12578529
3	8	3	2.87724587	0.87254745
1	8	4	5.86835055	0.94857282
2	8	4	-0.13724453	0.11108507
3	8	4	-1.36916241	0.77449485
1	8	5	3.16646483	0.96327142
2	8	5	0.36085608	0.11678117
3	8	5	0.07155036	0.78734269
1	8	6	0.00000000	0.00000000
2	8	6	0.00000000	0.00000000
3	8	6	0.00000000	0.00000000
1	8	7	1.86221092	1.09110375
2	8	7	0.15448539	0.12900793
3	8	7	-3.21739953	0.88735189

1	9	1	0.24838407	0.37280795
2	9	1	0.39131554	0.05193146
3	9	1	-1.60717443	0.36197503
1	9	2	0.00000000	0.00000000
2	9	2	0.00000000	0.00000000
3	9	2	0.00000000	0.00000000
1	9	3	2.85911549	0.66235692
2	9	3	0.48519698	0.08942432
3	9	3	-3.15993002	0.63114352
