# Supplementary material for: The genetic basis for the selection of dairy goats with enhanced resistance to gastrointestinal nematodes
Source: Parasite. 2017 Aug 9;24:32. doi: 10.1051/parasite/2017033 (PMC5550108; doi:10.1051/parasite/2017033)
Supplement: Supplementary file 2 — AIREML estimates MV4L [file parasite-24-32-s2.pdf]

AIREML estimates for effects in model MV4L

Traits are 1 = FECtrans, 2 = FAMACHA, 3 = PCV, 4 = lactation milk yield

Random effects in model are
1 = herd where GIN phenotypes were scored
2 = herd where lactation milk yield was recorded
7 = classifier for FAMACHA-scores

Final estimate for covariance matrix for effect 1

8.6720	0.31090	-2.1520	0.0000
0.31090	0.58560E-01	-0.72200E-01	0.0000
-2.1520	-0.72200E-01	2.6500	0.0000
0.0000	0.0000	0.0000	0.0000

Final estimate for covariance matrix for effect 2

0.0000	0.0000	0.0000	0.0000
0.0000	0.0000	0.0000	0.0000
0.0000	0.0000	0.0000	0.0000
0.0000	0.0000	0.0000	14800.

Final estimate for covariance matrix for effect 7

0.0000	0.0000	0.0000	0.0000
0.0000	0.17840E-01	0.0000	0.0000
0.0000	0.0000	0.0000	0.0000
0.0000	0.0000	0.0000	0.0000

Final estimate for residual covariance matrix

18.100	0.43060	-5.1660	12.570
0.43060	0.24680	-0.34030	1.6500
-5.1660	-0.34030	11.200	1.1360
12.570	1.6500	1.1360	7096.0

The solutions for the random effect 3 (animal) and 5 (permanent environment) are not listed below, their covariance matrices are:

Final estimate for covariance matrix for effect 3

1.8990	-0.88540E-02	-1.1110	24.020
-0.88540E-02	0.91690E-01	-0.36870	4.6850
-1.1110	-0.36870	4.1530	-73.100
24.020	4.6850	-73.100	8511.0

Final estimate for covariance matrix for effect 5

0.15490	0.86770E-02	0.29840	0.0000
0.86770E-02	0.85000E-03	0.18540E-01	0.0000
0.29840	0.18540E-01	0.63370	0.0000
0.0000	0.0000	0.0000	0.0000

Fixed effects in model are
4 = season (1=spring, 2=fall)
6 = breed (1=Saanen, 2=Alpine)

8 = type of anthelmintic (1=Eprinex, 2=missing, 3=Endex)
9 = FECRT (1=1%-20% reduction after treatment, 2=missing, 3=96%-100%, 4=81%-95%, 5=21%-40%, 6=61%-80%, 7=41%-60%)
10 = birth date (age class 1=291-980 days, 2=older than 980 days, 3=1-290 days)
11 = lactation (1=7th lactation, 2=4th lactation, 3=5th lactation, 4=9th lactation, 5=8th lactation, 6=6th lactation, 7=2nd lactation, 8=3rd lactation, 9=1st lactation)

trait	effect	level	solution	s.e.
1	1	1	-5.24586716	1.06863491
2	1	1	0.06292791	0.12058843
3	1	1	0.43166566	0.83213707
4	1	1	0.00000000	0.00000000
1	1	2	0.62185002	0.87837457
2	1	2	-0.13009646	0.09421124
3	1	2	0.14055003	0.64182898
4	1	2	0.00000000	0.00000000
1	1	3	1.24277572	0.92315609
2	1	3	0.13142956	0.10322850
3	1	3	-2.48883238	0.69139688
4	1	3	0.00000000	0.00000000
1	1	4	2.19217108	0.97793219
2	1	4	0.36081981	0.11288624
3	1	4	-0.56372133	0.76333367
4	1	4	0.00000000	0.00000000
1	1	5	4.08793745	0.91240225
2	1	5	0.00673178	0.09581558
3	1	5	-0.21532240	0.65488939
4	1	5	0.00000000	0.00000000
1	1	6	3.29889795	1.11022614
2	1	6	0.28402139	0.13291790
3	1	6	-0.27524126	0.91555483
4	1	6	0.00000000	0.00000000
1	1	7	-3.61197240	0.82711961
2	1	7	-0.36741764	0.09270148
3	1	7	2.25102064	0.63352018
4	1	7	0.00000000	0.00000000
1	1	8	1.04724988	0.97088639
2	1	8	0.26586573	0.11008231
3	1	8	2.65793430	0.75384694
4	1	8	0.00000000	0.00000000
1	1	9	1.12291532	1.00931439
2	1	9	0.06665984	0.11356860
3	1	9	-0.93750223	0.77272599
4	1	9	0.00000000	0.00000000
1	1	10	0.39240474	0.99562590
2	1	10	-0.01556357	0.11325142
3	1	10	2.17511787	0.77710132
4	1	10	0.00000000	0.00000000
1	1	11	1.55151045	0.94347104
2	1	11	0.25866982	0.11540567
3	1	11	-0.94074876	0.79240468
4	1	11	0.00000000	0.00000000

1	1	12	-0.11269605	0.96026406
2	1	12	-0.45719686	0.11353396
3	1	12	-1.13470375	0.77755899
4	1	12	0.00000000	0.00000000
1	1	13	4.28683610	0.95522416
2	1	13	0.18288600	0.10695472
3	1	13	-1.41206905	0.73288481
4	1	13	0.00000000	0.00000000
1	1	14	-2.80628006	0.91959747
2	1	14	0.15335221	0.10744610
3	1	14	0.51630528	0.73744923
4	1	14	0.00000000	0.00000000
1	1	15	1.33588348	0.93035991
2	1	15	-0.03110245	0.10304079
3	1	15	-0.04706389	0.70414853
4	1	15	0.00000000	0.00000000
1	1	16	2.15916017	0.84736262
2	1	16	-0.07335994	0.09141627
3	1	16	-1.27628991	0.62635729
4	1	16	0.00000000	0.00000000
1	1	17	-0.96812802	0.86072405
2	1	17	-0.29648560	0.09361018
3	1	17	-1.26346050	0.64352695
4	1	17	0.00000000	0.00000000
1	1	18	-2.62549975	0.91277900
2	1	18	-0.15414934	0.10851272
3	1	18	0.42971552	0.74181515
4	1	18	0.00000000	0.00000000
1	1	19	-3.04031963	0.85709602
2	1	19	-0.01313701	0.09595575
3	1	19	0.56720133	0.65250866
4	1	19	0.00000000	0.00000000
1	1	20	-4.92882928	0.85549597
2	1	20	-0.23485518	0.09419454
3	1	20	1.38544483	0.64600527
4	1	20	0.00000000	0.00000000
1	2	1	0.00000000	0.00000000
2	2	1	0.00000000	0.00000000
3	2	1	0.00000000	0.00000000
4	2	1	-55.98053329	44.99102845
1	2	2	0.00000000	0.00000000
2	2	2	0.00000000	0.00000000
3	2	2	0.00000000	0.00000000
4	2	2	55.24192869	38.21318170
1	2	3	0.00000000	0.00000000
2	2	3	0.00000000	0.00000000
3	2	3	0.00000000	0.00000000
4	2	3	-112.33040319	39.82692836
1	2	4	0.00000000	0.00000000
2	2	4	0.00000000	0.00000000
3	2	4	0.00000000	0.00000000
4	2	4	-167.18971869	44.05718717
1	2	5	0.00000000	0.00000000
2	2	5	0.00000000	0.00000000

3	2	5	0.00000000	0.00000000
4	2	5	81.79544630	42.01821139
1	2	6	0.00000000	0.00000000
2	2	6	0.00000000	0.00000000
3	2	6	0.00000000	0.00000000
4	2	6	72.52646884	50.63536130
1	2	7	0.00000000	0.00000000
2	2	7	0.00000000	0.00000000
3	2	7	0.00000000	0.00000000
4	2	7	24.98185130	37.02733904
1	2	8	0.00000000	0.00000000
2	2	8	0.00000000	0.00000000
3	2	8	0.00000000	0.00000000
4	2	8	16.70520424	48.52979182
1	2	9	0.00000000	0.00000000
2	2	9	0.00000000	0.00000000
3	2	9	0.00000000	0.00000000
4	2	9	25.51877841	41.01035622
1	2	10	0.00000000	0.00000000
2	2	10	0.00000000	0.00000000
3	2	10	0.00000000	0.00000000
4	2	10	-113.43333879	43.79265007
1	2	11	0.00000000	0.00000000
2	2	11	0.00000000	0.00000000
3	2	11	0.00000000	0.00000000
4	2	11	-83.59821480	41.75486928
1	2	12	0.00000000	0.00000000
2	2	12	0.00000000	0.00000000
3	2	12	0.00000000	0.00000000
4	2	12	-34.25388946	43.05716628
1	2	13	0.00000000	0.00000000
2	2	13	0.00000000	0.00000000
3	2	13	0.00000000	0.00000000
4	2	13	-166.66303538	40.11849316
1	2	14	0.00000000	0.00000000
2	2	14	0.00000000	0.00000000
3	2	14	0.00000000	0.00000000
4	2	14	70.71126145	40.33278032
1	2	15	0.00000000	0.00000000
2	2	15	0.00000000	0.00000000
3	2	15	0.00000000	0.00000000
4	2	15	-145.18570013	35.91482996
1	2	16	0.00000000	0.00000000
2	2	16	0.00000000	0.00000000
3	2	16	0.00000000	0.00000000
4	2	16	203.75083536	41.05075151
1	2	17	0.00000000	0.00000000
2	2	17	0.00000000	0.00000000
3	2	17	0.00000000	0.00000000
4	2	17	-43.16388776	40.84591480
1	2	18	0.00000000	0.00000000
2	2	18	0.00000000	0.00000000
3	2	18	0.00000000	0.00000000
4	2	18	156.26682165	36.82143937

1	2	19	0.00000000	0.00000000
2	2	19	0.00000000	0.00000000
3	2	19	0.00000000	0.00000000
4	2	19	214.30012527	38.99040993
1	4	1	10.31679784	1.14655536
2	4	1	2.76213288	0.18456701
3	4	1	28.72880271	0.86730972
4	4	1	0.00000000	0.00000000
1	4	2	10.62323531	1.20312801
2	4	2	3.10693011	0.15645907
3	4	2	29.40153006	0.92034763
4	4	2	0.00000000	0.00000000
1	6	1	0.00000000	0.00000000
2	6	1	0.00000000	0.00000000
3	6	1	0.00000000	0.00000000
4	6	1	0.00000000	0.00000000
1	6	2	0.55682470	0.79105730
2	6	2	0.39762386	0.10413911
3	6	2	-1.03782460	0.71681902
4	6	2	-49.29831235	38.88338592
1	7	1	0.00000000	0.00000000
2	7	1	0.00000000	0.13356646
3	7	1	0.00000000	0.00000000
4	7	1	0.00000000	0.00000000
1	7	2	0.00000000	0.00000000
2	7	2	0.15162396	0.08268725
3	7	2	0.00000000	0.00000000
4	7	2	0.00000000	0.00000000
1	7	3	0.00000000	0.00000000
2	7	3	-0.05967388	0.08693327
3	7	3	0.00000000	0.00000000
4	7	3	0.00000000	0.00000000
1	7	4	0.00000000	0.00000000
2	7	4	-0.09195008	0.08909307
3	7	4	0.00000000	0.00000000
4	7	4	0.00000000	0.00000000
1	8	1	0.82617485	0.73057240
2	8	1	0.26787127	0.08892447
3	8	1	0.04456736	0.59519348
4	8	1	0.00000000	0.00000000
1	8	2	0.00000000	0.00000000
2	8	2	0.00000000	0.00000000
3	8	2	0.00000000	0.00000000
4	8	2	0.00000000	0.00000000
1	8	3	0.26837726	0.59155388
2	8	3	0.20296401	0.07225709
3	8	3	-2.60730067	0.49746642
4	8	3	0.00000000	0.00000000
1	9	1	-4.00884480	0.95585761
2	9	1	-0.31925599	0.11731679
3	9	1	-1.89048852	0.75831512
4	9	1	0.00000000	0.00000000
1	9	2	0.26976554	0.73576118
2	9	2	-0.02485450	0.08562186

3	9	2	0.50697603	0.58075430
4	9	2	0.00000000	0.00000000
1	9	3	0.64143638	0.72320093
2	9	3	-0.16273465	0.08575880
3	9	3	2.84981967	0.57033572
4	9	3	0.00000000	0.00000000
1	9	4	2.69828789	0.53289822
2	9	4	-0.49560208	0.07050530
3	9	4	-1.43865108	0.42075256
4	9	4	0.00000000	0.00000000
1	9	5	0.00000000	0.00000000
2	9	5	0.00000000	0.00000000
3	9	5	0.00000000	0.00000000
4	9	5	0.00000000	0.00000000
1	9	6	-3.14201202	0.96274338
2	9	6	-0.35276116	0.11686685
3	9	6	-0.12417322	0.75709263
4	9	6	0.00000000	0.00000000
1	9	7	-1.31383212	0.75845141
2	9	7	-0.20648054	0.08816316
3	9	7	-3.25804192	0.59409316
4	9	7	0.00000000	0.00000000
1	10	1	0.24573206	0.37233150
2	10	1	0.39374744	0.05191179
3	10	1	-1.59934985	0.36060119
4	10	1	0.00000000	0.00000000
1	10	2	0.00000000	0.00000000
2	10	2	0.00000000	0.00000000
3	10	2	0.00000000	0.00000000
4	10	2	0.00000000	0.00000000
1	10	3	2.84039754	0.66144017
2	10	3	0.48335337	0.08940144
3	10	3	-3.11081181	0.62559918
4	10	3	0.00000000	0.00000000
1	11	1	0.00000000	0.00000000
2	11	1	0.00000000	0.00000000
3	11	1	0.00000000	0.00000000
4	11	1	729.28970991	44.01495087
1	11	2	0.00000000	0.00000000
2	11	2	0.00000000	0.00000000
3	11	2	0.00000000	0.00000000
4	11	2	753.10227568	41.44900839
1	11	3	0.00000000	0.00000000
2	11	3	0.00000000	0.00000000
3	11	3	0.00000000	0.00000000
4	11	3	743.79878954	42.09434490
1	11	4	0.00000000	0.00000000
2	11	4	0.00000000	0.00000000
3	11	4	0.00000000	0.00000000
4	11	4	695.30574314	61.01449265
1	11	5	0.00000000	0.00000000
2	11	5	0.00000000	0.00000000
3	11	5	0.00000000	0.00000000
4	11	5	711.48520316	47.93582279

1	11	6	0.00000000	0.00000000
2	11	6	0.00000000	0.00000000
3	11	6	0.00000000	0.00000000
4	11	6	775.39555831	44.19500584
1	11	7	0.00000000	0.00000000
2	11	7	0.00000000	0.00000000
3	11	7	0.00000000	0.00000000
4	11	7	677.16661064	41.41860205
1	11	8	0.00000000	0.00000000
2	11	8	0.00000000	0.00000000
3	11	8	0.00000000	0.00000000
4	11	8	707.83200845	41.21073785
1	11	9	0.00000000	0.00000000
2	11	9	0.00000000	0.00000000
3	11	9	0.00000000	0.00000000
4	11	9	530.44963842	41.34647608
