# Supplementary material for: The genetic basis for the selection of dairy goats with enhanced resistance to gastrointestinal nematodes
Source: Parasite. 2017 Aug 9;24:32. doi: 10.1051/parasite/2017033 (PMC5550108; doi:10.1051/parasite/2017033)
Supplement: Supplementary file 3 — AIREML estimates MV4TD [file parasite-24-32-s3.pdf]

AIREML estimates for effects in model MV4TD

Traits are 1 = FECtrans, 2 = FAMACHA, 3 = PCV, 4 = Test-day milk yield

Random effects in model are
1 = herd where GIN phenotypes were scored
2 = herd where closest milk test day yield was recorded
7 = classifier for FAMACHA-scores

Final estimate for covariance matrix for effect 1

8.8188	0.31128	-1.9201	0.0000
0.31128	0.59503E-01	-0.37185E-01	0.0000
-1.9201	-0.37185E-01	2.0746	0.0000
0.0000	0.0000	0.0000	0.0000

Final estimate for covariance matrix for effect 2

0.0000	0.0000	0.0000	0.0000
0.0000	0.0000	0.0000	0.0000
0.0000	0.0000	0.0000	0.0000
0.0000	0.0000	0.0000	0.27261

Final estimate for covariance matrix for effect 7

0.0000	0.0000	0.0000	0.0000
0.0000	0.22500E-01	0.0000	0.0000
0.0000	0.0000	0.0000	0.0000
0.0000	0.0000	0.0000	0.0000

Final estimate for residual covariance matrix

17.988	0.42806	-5.6971	-0.47130
0.42806	0.24627	-0.36188	0.34734E-02
-5.6971	-0.36188	12.175	0.12860
-0.47130	0.34734E-02	0.12860	0.35391

The solutions for the random effect 3 (animal) and 5 (permanent environment) are not listed below, their covariance matrices are:

Final estimate for covariance matrix for effect 3

2.0361	-0.10289E-01	-0.93807	0.20606
-0.10289E-01	0.91444E-01	-0.34744	0.93344E-02
-0.93807	-0.34744	3.6332	-0.19859
0.20606	0.93344E-02	-0.19859	0.87697E-01

Final estimate for covariance matrix for effect 5

0.19396	0.69981E-02	0.33203	0.31906E-01
0.69981E-02	0.63299E-03	0.16170E-01	0.45395E-03
0.33203	0.16170E-01	0.61401	0.47131E-01
0.31906E-01	0.45395E-03	0.47131E-01	0.64844E-02

Fixed effects in model are
4 = season (1=spring, 2=fall)
6 = breed (1=Saanen, 2=Alpine)

8 = type of anthelmintic (1=Eprinex, 2=missing, 3=Endex)
9 = FECRT (1=1%-20% reduction after treatment, 2=missing, 3=96%-100%, 4=81%-95%, 5=21%-40%, 6=61%-80%, 7=41%-60%)
10 = birth date (age class 1=291-980 days, 2=older than 980 days, 3=1-290 days)
11 = lactation (1=6ff lactation, 2=5th lactation, 3=4th lactation, 4=2nd lactation, 5=3rd lactation, 6=1st lactation)
12 = lactation stage (month in lactation, 1=4, 2=6-7, 3=3, 4=5, 5=8, 6=>8, 7=0-2)

trait	effect	level	solution	s.e.
1	1	1	-5.41368084	1.07377815
2	1	1	0.05511732	0.12003894
3	1	1	0.53579139	0.77772574
4	1	1	0.00000000	0.00000000
1	1	2	0.58294573	0.88271006
2	1	2	-0.12864400	0.09414121
3	1	2	0.12600848	0.60753885
4	1	2	0.00000000	0.00000000
1	1	3	1.37618333	0.92938414
2	1	3	0.12807171	0.10311341
3	1	3	-2.39517344	0.64749624
4	1	3	0.00000000	0.00000000
1	1	4	2.24031069	0.98611690
2	1	4	0.36037599	0.11246917
3	1	4	-0.48572555	0.70975982
4	1	4	0.00000000	0.00000000
1	1	5	4.09996153	0.91826890
2	1	5	0.00849712	0.09571850
3	1	5	-0.21247232	0.61369021
4	1	5	0.00000000	0.00000000
1	1	6	3.42485196	1.12118461
2	1	6	0.28187377	0.13188676
3	1	6	-0.24540859	0.84305620
4	1	6	0.00000000	0.00000000
1	1	7	-3.50460329	0.83383000
2	1	7	-0.35919104	0.09257070
3	1	7	2.06460980	0.58865951
4	1	7	0.00000000	0.00000000
1	1	8	0.96859173	0.97762496
2	1	8	0.27834502	0.10965592
3	1	8	2.56874567	0.70955661
4	1	8	0.00000000	0.00000000
1	1	9	1.29889119	1.01734945
2	1	9	0.06484250	0.11321179
3	1	9	-0.93237821	0.72386410
4	1	9	0.00000000	0.00000000
1	1	10	0.49330277	1.00086835
2	1	10	-0.00395226	0.11277874
3	1	10	2.04490779	0.73082226
4	1	10	0.00000000	0.00000000
1	1	11	1.51019483	0.95306790
2	1	11	0.25040960	0.11465458
3	1	11	-0.83658968	0.73271271

4	1	11	0.00000000	0.00000000
1	1	12	-0.12800986	0.96787022
2	1	12	-0.46343090	0.11291846
3	1	12	-1.13079212	0.72392937
4	1	12	0.00000000	0.00000000
1	1	13	4.25288565	0.96033419
2	1	13	0.18143759	0.10664050
3	1	13	-1.33399431	0.68984788
4	1	13	0.00000000	0.00000000
1	1	14	-2.69822054	0.92572002
2	1	14	0.15450702	0.10704105
3	1	14	0.57914440	0.68791750
4	1	14	0.00000000	0.00000000
1	1	15	1.29900452	0.93619122
2	1	15	-0.03102601	0.10280006
3	1	15	-0.07171830	0.66382213
4	1	15	0.00000000	0.00000000
1	1	16	2.26957083	0.85261268
2	1	16	-0.07819419	0.09136039
3	1	16	-1.33903595	0.58770694
4	1	16	0.00000000	0.00000000
1	1	17	-1.13317292	0.86586175
2	1	17	-0.29927623	0.09349505
3	1	17	-1.29954649	0.60342939
4	1	17	0.00000000	0.00000000
1	1	18	-2.74030730	0.92028858
2	1	18	-0.14831225	0.10801888
3	1	18	0.38794287	0.68902892
4	1	18	0.00000000	0.00000000
1	1	19	-3.27655571	0.86255389
2	1	19	-0.01591543	0.09583917
3	1	19	0.63319461	0.60956758
4	1	19	0.00000000	0.00000000
1	1	20	-4.92214432	0.86116097
2	1	20	-0.23553534	0.09406162
3	1	20	1.34248996	0.60388605
4	1	20	0.00000000	0.00000000
1	2	1	0.00000000	0.00000000
2	2	1	0.00000000	0.00000000
3	2	1	0.00000000	0.00000000
4	2	1	-0.05646081	0.18720061
1	2	2	0.00000000	0.00000000
2	2	2	0.00000000	0.00000000
3	2	2	0.00000000	0.00000000
4	2	2	-0.40170201	0.16621828
1	2	3	0.00000000	0.00000000
2	2	3	0.00000000	0.00000000
3	2	3	0.00000000	0.00000000
4	2	3	-0.57807279	0.18177859
1	2	4	0.00000000	0.00000000
2	2	4	0.00000000	0.00000000
3	2	4	0.00000000	0.00000000
4	2	4	0.57298931	0.15239386
1	2	5	0.00000000	0.00000000

2	2	5	0.00000000	0.00000000
3	2	5	0.00000000	0.00000000
4	2	5	0.46811473	0.20255332
1	2	6	0.00000000	0.00000000
2	2	6	0.00000000	0.00000000
3	2	6	0.00000000	0.00000000
4	2	6	-0.11817549	0.15286420
1	2	7	0.00000000	0.00000000
2	2	7	0.00000000	0.00000000
3	2	7	0.00000000	0.00000000
4	2	7	-0.31087570	0.23834426
1	2	8	0.00000000	0.00000000
2	2	8	0.00000000	0.00000000
3	2	8	0.00000000	0.00000000
4	2	8	0.28795114	0.17025240
1	2	9	0.00000000	0.00000000
2	2	9	0.00000000	0.00000000
3	2	9	0.00000000	0.00000000
4	2	9	-0.65900770	0.17307387
1	2	10	0.00000000	0.00000000
2	2	10	0.00000000	0.00000000
3	2	10	0.00000000	0.00000000
4	2	10	0.07359581	0.17790006
1	2	11	0.00000000	0.00000000
2	2	11	0.00000000	0.00000000
3	2	11	0.00000000	0.00000000
4	2	11	-0.14373124	0.17091593
1	2	12	0.00000000	0.00000000
2	2	12	0.00000000	0.00000000
3	2	12	0.00000000	0.00000000
4	2	12	-0.50415038	0.16679230
1	2	13	0.00000000	0.00000000
2	2	13	0.00000000	0.00000000
3	2	13	0.00000000	0.00000000
4	2	13	-0.07259793	0.16639096
1	2	14	0.00000000	0.00000000
2	2	14	0.00000000	0.00000000
3	2	14	0.00000000	0.00000000
4	2	14	-0.63926804	0.15042652
1	2	15	0.00000000	0.00000000
2	2	15	0.00000000	0.00000000
3	2	15	0.00000000	0.00000000
4	2	15	0.71917665	0.16562823
1	2	16	0.00000000	0.00000000
2	2	16	0.00000000	0.00000000
3	2	16	0.00000000	0.00000000
4	2	16	-0.29604164	0.15719696
1	2	17	0.00000000	0.00000000
2	2	17	0.00000000	0.00000000
3	2	17	0.00000000	0.00000000
4	2	17	0.92888192	0.15357341
1	2	18	0.00000000	0.00000000
2	2	18	0.00000000	0.00000000
3	2	18	0.00000000	0.00000000

4	2	18	0.72937417	0.15926530
1	4	1	10.61285430	1.21420394
2	4	1	2.96891834	0.20408661
3	4	1	26.07599028	0.92623418
4	4	1	0.00000000	0.00000000
1	4	2	10.80424254	1.12295131
2	4	2	3.30596770	0.15235286
3	4	2	26.80081997	0.82738198
4	4	2	0.00000000	0.00000000
1	6	1	0.00000000	0.00000000
2	6	1	0.00000000	0.00000000
3	6	1	0.00000000	0.00000000
4	6	1	0.00000000	0.00000000
1	6	2	0.63553863	0.79752260
2	6	2	0.39019111	0.10376614
3	6	2	-0.97793459	0.67790606
4	6	2	-0.01685417	0.15104271
1	7	1	0.00000000	0.00000000
2	7	1	0.00000000	0.15000097
3	7	1	0.00000000	0.00000000
4	7	1	0.00000000	0.00000000
1	7	2	0.00000000	0.00000000
2	7	2	0.16218901	0.09166622
3	7	2	0.00000000	0.00000000
4	7	2	0.00000000	0.00000000
1	7	3	0.00000000	0.00000000
2	7	3	-0.05968109	0.09571493
3	7	3	0.00000000	0.00000000
4	7	3	0.00000000	0.00000000
1	7	4	0.00000000	0.00000000
2	7	4	-0.10250792	0.09777471
3	7	4	0.00000000	0.00000000
4	7	4	0.00000000	0.00000000
1	8	1	0.67722104	0.59321565
2	8	1	0.06831353	0.07160935
3	8	1	2.59580274	0.49173204
4	8	1	0.00000000	0.00000000
1	8	2	-0.42816931	0.58589153
2	8	2	-0.20423938	0.07221320
3	8	2	2.67312492	0.51154900
4	8	2	0.00000000	0.00000000
1	8	3	0.00000000	0.00000000
2	8	3	0.00000000	0.00000000
3	8	3	0.00000000	0.00000000
4	8	3	0.00000000	0.00000000
1	9	1	-3.97059748	0.94226381
2	9	1	-0.32443148	0.11727738
3	9	1	-1.92910441	0.78408395
4	9	1	0.00000000	0.00000000
1	9	2	0.41924699	0.72404972
2	9	2	-0.02355443	0.08551958
3	9	2	0.42914273	0.60025283
4	9	2	0.00000000	0.00000000
1	9	3	0.42985177	0.71173882

2	9	3	−0.15800828	0.08570298
3	9	3	2.85787956	0.58954325
4	9	3	0.00000000	0.00000000
1	9	4	2.47103613	0.52544393
2	9	4	−0.49524717	0.07064502
3	9	4	−1.38739194	0.43572576
4	9	4	0.00000000	0.00000000
1	9	5	0.00000000	0.00000000
2	9	5	0.00000000	0.00000000
3	9	5	0.00000000	0.00000000
4	9	5	0.00000000	0.00000000
1	9	6	−3.18980601	0.95848554
2	9	6	−0.36188526	0.11683933
3	9	6	−0.06838854	0.78410893
4	9	6	0.00000000	0.00000000
1	9	7	−1.45558358	0.75047069
2	9	7	−0.20545505	0.08805680
3	9	7	−3.24870015	0.61461739
4	9	7	0.00000000	0.00000000
1	10	1	0.22050943	0.37385401
2	10	1	0.38907204	0.05189806
3	10	1	−1.58470816	0.36140733
4	10	1	0.00000000	0.00000000
1	10	2	0.00000000	0.00000000
2	10	2	0.00000000	0.00000000
3	10	2	0.00000000	0.00000000
4	10	2	0.00000000	0.00000000
1	10	3	2.72948385	0.66343075
2	10	3	0.49155768	0.08934029
3	10	3	−3.15098441	0.63068012
4	10	3	0.00000000	0.00000000
1	11	1	0.00000000	0.00000000
2	11	1	0.00000000	0.00000000
3	11	1	0.00000000	0.00000000
4	11	1	1.70412879	0.17585780
1	11	2	0.00000000	0.00000000
2	11	2	0.00000000	0.00000000
3	11	2	0.00000000	0.00000000
4	11	2	1.88407759	0.17748820
1	11	3	0.00000000	0.00000000
2	11	3	0.00000000	0.00000000
3	11	3	0.00000000	0.00000000
4	11	3	1.87905725	0.17408026
1	11	4	0.00000000	0.00000000
2	11	4	0.00000000	0.00000000
3	11	4	0.00000000	0.00000000
4	11	4	1.66821859	0.17427293
1	11	5	0.00000000	0.00000000
2	11	5	0.00000000	0.00000000
3	11	5	0.00000000	0.00000000
4	11	5	1.83095831	0.17302610
1	11	6	0.00000000	0.00000000
2	11	6	0.00000000	0.00000000
3	11	6	0.00000000	0.00000000

4	11	6	1.15446679	0.17378657
1	12	1	0.00000000	0.00000000
2	12	1	0.00000000	0.00000000
3	12	1	0.00000000	0.00000000
4	12	1	1.24339037	0.05222125
1	12	2	0.00000000	0.00000000
2	12	2	0.00000000	0.00000000
3	12	2	0.00000000	0.00000000
4	12	2	0.25766878	0.05419215
1	12	3	0.00000000	0.00000000
2	12	3	0.00000000	0.00000000
3	12	3	0.00000000	0.00000000
4	12	3	1.46960453	0.05821547
1	12	4	0.00000000	0.00000000
2	12	4	0.00000000	0.00000000
3	12	4	0.00000000	0.00000000
4	12	4	1.15775728	0.04915970
1	12	5	0.00000000	0.00000000
2	12	5	0.00000000	0.00000000
3	12	5	0.00000000	0.00000000
4	12	5	0.00000000	0.00000000
1	12	6	0.00000000	0.00000000
2	12	6	0.00000000	0.00000000
3	12	6	0.00000000	0.00000000
4	12	6	-0.00976060	0.05325555
1	12	7	0.00000000	0.00000000
2	12	7	0.00000000	0.00000000
3	12	7	0.00000000	0.00000000
4	12	7	1.36909347	0.07670242
