# Supplementary material for: The genetic basis for the selection of dairy goats with enhanced resistance to gastrointestinal nematodes
Source: Parasite. 2017 Aug 9;24:32. doi: 10.1051/parasite/2017033 (PMC5550108; doi:10.1051/parasite/2017033)
Supplement: Supplementary file 4 — AIREML estimates MV4TD for FECtrans_haem [file parasite-24-32-s4.pdf]

AIREML estimates for effects in model FECtrans_haem

Traits are 1 = FECtrans_haem, 2 = FAMACHA, 3 = PCV, 4 = Test-day milk yield

Random effects in model are
1 = herd where GIN phenotypes were scored
2 = herd where closest milk test day yield was recorded
7 = classifier for FAMACHA-scores

Final estimate for covariance matrix for effect 1

8.0818	0.24053	-1.6409	0.0000
0.24053	0.49136E-01	-0.93793E-01	0.0000
-1.6409	-0.93793E-01	2.5137	0.0000
0.0000	0.0000	0.0000	0.0000

Final estimate for covariance matrix for effect 2

0.0000	0.0000	0.0000	0.0000
0.0000	0.0000	0.0000	0.0000
0.0000	0.0000	0.0000	0.0000
0.0000	0.0000	0.0000	0.26950

Final estimate for covariance matrix for effect 7

0.0000	0.0000	0.0000	0.0000
0.0000	0.43621E-01	0.0000	0.0000
0.0000	0.0000	0.0000	0.0000
0.0000	0.0000	0.0000	0.0000

Final estimate for residual covariance matrix

15.259	0.33491	-4.9159	-0.10074
0.33491	0.23163	-0.25322	0.87630E-02
-4.9159	-0.25322	12.556	0.11050
-0.10074	0.87630E-02	0.11050	0.35758

The solutions for the random effect 3 (animal) and 5 (permanent environment) are not listed below, their covariance matrices are:

Final estimate for covariance matrix for effect 3

0.80901	0.62686E-01	-1.0052	0.13561
0.62686E-01	0.10025	-0.39677	0.10845E-01
-1.0052	-0.39677	3.9964	-0.17965
0.13561	0.10845E-01	-0.17965	0.84879E-01

Final estimate for covariance matrix for effect 5

0.18007E-01	-0.31785E-03	0.30270E-01	0.56762E-02
-0.31785E-03	0.61102E-03	0.48225E-02	-0.47409E-03
0.30270E-01	0.48225E-02	0.97840E-01	0.62978E-02
0.56762E-02	-0.47409E-03	0.62978E-02	0.20135E-02

Fixed effects in model are
4 = season (1=spring, 2=fall)
6 = breed (1=Saanen, 2=Alpine)

8 = type of anthelmintic (1=Eprinex, 2=missing, 3=Endex)
9 = FECRT (1=0% reduction after treatment, 2=missing, 3=96%-100%, 4=1%-20%, 5=21%-40%)
10 = birth date (age class 1=291-980 days, 2=older than 980 days, 3=1-290 days)
11 = lactation (1=6ff lactation, 2=5th lactation, 3=4th lactation, 4=2nd lactation, 5=3rd lactation, 6=1st lactation)
12 = lactation stage (month in lactation, 1=4 month, 2=6-7, 3=3, 4=5, 5=8, 6=>8, 7=0-2)

trait	effect	level	solution	s.e.
1	1	1	-3.77921495	0.99402240
2	1	1	0.07921332	0.11276837
3	1	1	-0.33073650	0.79892663
4	1	1	0.00000000	0.00000000
1	1	2	3.02267277	0.81876410
2	1	2	-0.08139104	0.08771984
3	1	2	0.59059435	0.61822370
4	1	2	0.00000000	0.00000000
1	1	3	1.92957700	0.88245611
2	1	3	0.20448374	0.10023460
3	1	3	-2.28828970	0.69598785
4	1	3	0.00000000	0.00000000
1	1	4	2.44812396	0.91660643
2	1	4	0.33884819	0.10971625
3	1	4	-0.95919213	0.76714239
4	1	4	0.00000000	0.00000000
1	1	5	3.92981439	0.87152141
2	1	5	0.05085477	0.09165421
3	1	5	-0.06194467	0.64946831
4	1	5	0.00000000	0.00000000
1	1	6	3.72994377	1.01874143
2	1	6	0.28639104	0.12971450
3	1	6	-0.06906744	0.92832631
4	1	6	0.00000000	0.00000000
1	1	7	-5.38697279	0.79599021
2	1	7	-0.36365141	0.08966931
3	1	7	2.69957649	0.63915297
4	1	7	0.00000000	0.00000000
1	1	8	-1.87952497	0.85593099
2	1	8	0.22215503	0.10163647
3	1	8	3.16915805	0.72005479
4	1	8	0.00000000	0.00000000
1	1	9	2.59006047	0.95156357
2	1	9	0.01919839	0.11012262
3	1	9	-0.83233683	0.77863738
4	1	9	0.00000000	0.00000000
1	1	10	2.10950418	0.90591821
2	1	10	-0.14377543	0.10763685
3	1	10	1.00748539	0.76500928
4	1	10	0.00000000	0.00000000
1	1	11	-3.95454411	0.89968625
2	1	11	0.09211203	0.11447410
3	1	11	-1.26068556	0.81629314

4	1	11	0.00000000	0.00000000
1	1	12	-2.31641644	0.90856505
2	1	12	-0.39456726	0.11192350
3	1	12	-0.43040159	0.79594568
4	1	12	0.00000000	0.00000000
1	1	13	2.24324594	0.86894621
2	1	13	0.11581379	0.10156128
3	1	13	-2.20771375	0.71826638
4	1	13	0.00000000	0.00000000
1	1	14	-1.00865342	0.85699045
2	1	14	0.09353352	0.10442134
3	1	14	0.33620554	0.74039223
4	1	14	0.00000000	0.00000000
1	1	15	-0.69128258	0.85325409
2	1	15	-0.00132523	0.09622039
3	1	15	-0.92644187	0.68274419
4	1	15	0.00000000	0.00000000
1	1	16	3.33868246	0.81262323
2	1	16	-0.07217327	0.08761949
3	1	16	-0.57064236	0.62496531
4	1	16	0.00000000	0.00000000
1	1	17	-0.15987917	0.80816158
2	1	17	-0.21189005	0.08823563
3	1	17	-1.84895209	0.63355238
4	1	17	0.00000000	0.00000000
1	1	18	-0.13551515	0.84878108
2	1	18	-0.08506642	0.10522757
3	1	18	0.74953159	0.74375038
4	1	18	0.00000000	0.00000000
1	1	19	-1.03705176	0.81210745
2	1	19	0.10741148	0.09180176
3	1	19	0.96321774	0.65163275
4	1	19	0.00000000	0.00000000
1	1	20	-4.99256961	0.85042365
2	1	20	-0.25617520	0.09432694
3	1	20	2.27063537	0.67252570
4	1	20	0.00000000	0.00000000
1	2	1	0.00000000	0.00000000
2	2	1	0.00000000	0.00000000
3	2	1	0.00000000	0.00000000
4	2	1	-0.05827368	0.18680840
1	2	2	0.00000000	0.00000000
2	2	2	0.00000000	0.00000000
3	2	2	0.00000000	0.00000000
4	2	2	-0.39423268	0.16587714
1	2	3	0.00000000	0.00000000
2	2	3	0.00000000	0.00000000
3	2	3	0.00000000	0.00000000
4	2	3	-0.58733602	0.18121739
1	2	4	0.00000000	0.00000000
2	2	4	0.00000000	0.00000000
3	2	4	0.00000000	0.00000000
4	2	4	0.56432740	0.15214116
1	2	5	0.00000000	0.00000000

2	2	5	0.00000000	0.00000000
3	2	5	0.00000000	0.00000000
4	2	5	0.47196970	0.20180120
1	2	6	0.00000000	0.00000000
2	2	6	0.00000000	0.00000000
3	2	6	0.00000000	0.00000000
4	2	6	-0.11612665	0.15255256
1	2	7	0.00000000	0.00000000
2	2	7	0.00000000	0.00000000
3	2	7	0.00000000	0.00000000
4	2	7	-0.29818167	0.23819876
1	2	8	0.00000000	0.00000000
2	2	8	0.00000000	0.00000000
3	2	8	0.00000000	0.00000000
4	2	8	0.28459408	0.16980865
1	2	9	0.00000000	0.00000000
2	2	9	0.00000000	0.00000000
3	2	9	0.00000000	0.00000000
4	2	9	-0.66118662	0.17251151
1	2	10	0.00000000	0.00000000
2	2	10	0.00000000	0.00000000
3	2	10	0.00000000	0.00000000
4	2	10	0.05613105	0.17746973
1	2	11	0.00000000	0.00000000
2	2	11	0.00000000	0.00000000
3	2	11	0.00000000	0.00000000
4	2	11	-0.13210888	0.17036505
1	2	12	0.00000000	0.00000000
2	2	12	0.00000000	0.00000000
3	2	12	0.00000000	0.00000000
4	2	12	-0.50936311	0.16646424
1	2	13	0.00000000	0.00000000
2	2	13	0.00000000	0.00000000
3	2	13	0.00000000	0.00000000
4	2	13	-0.07253747	0.16604868
1	2	14	0.00000000	0.00000000
2	2	14	0.00000000	0.00000000
3	2	14	0.00000000	0.00000000
4	2	14	-0.63436824	0.15020155
1	2	15	0.00000000	0.00000000
2	2	15	0.00000000	0.00000000
3	2	15	0.00000000	0.00000000
4	2	15	0.71516605	0.16517609
1	2	16	0.00000000	0.00000000
2	2	16	0.00000000	0.00000000
3	2	16	0.00000000	0.00000000
4	2	16	-0.29878804	0.15694714
1	2	17	0.00000000	0.00000000
2	2	17	0.00000000	0.00000000
3	2	17	0.00000000	0.00000000
4	2	17	0.93735189	0.15334546
1	2	18	0.00000000	0.00000000
2	2	18	0.00000000	0.00000000
3	2	18	0.00000000	0.00000000

4	2	18	0.73296288	0.15899759
1	4	1	9.24016367	0.96711842
2	4	1	2.88069926	0.23153282
3	4	1	27.77551531	0.79664461
4	4	1	0.00000000	0.00000000
1	4	2	9.26064580	0.86598716
2	4	2	3.16629543	0.15030312
3	4	2	28.97347094	0.66917248
4	4	2	0.00000000	0.00000000
1	6	1	0.00000000	0.00000000
2	6	1	0.00000000	0.00000000
3	6	1	0.00000000	0.00000000
4	6	1	0.00000000	0.00000000
1	6	2	0.21583987	0.68773548
2	6	2	0.41394676	0.10237030
3	6	2	-1.38661110	0.72128492
4	6	2	-0.01027296	0.15011401
1	7	1	0.00000000	0.00000000
2	7	1	0.00000000	0.20199010
3	7	1	0.00000000	0.00000000
4	7	1	0.00000000	0.00000000
1	7	2	0.00000000	0.00000000
2	7	2	0.12017247	0.12070468
3	7	2	0.00000000	0.00000000
4	7	2	0.00000000	0.00000000
1	7	3	0.00000000	0.00000000
2	7	3	0.10665460	0.12227190
3	7	3	0.00000000	0.00000000
4	7	3	0.00000000	0.00000000
1	7	4	0.00000000	0.00000000
2	7	4	-0.22682707	0.12493385
3	7	4	0.00000000	0.00000000
4	7	4	0.00000000	0.00000000
1	8	1	5.96426398	0.62204666
2	8	1	-0.15071453	0.08240892
3	8	1	-2.72712796	0.56043058
4	8	1	0.00000000	0.00000000
1	8	2	-1.03555544	0.46881182
2	8	2	-0.15521972	0.06638282
3	8	2	1.54722194	0.45324001
4	8	2	0.00000000	0.00000000
1	8	3	0.00000000	0.00000000
2	8	3	0.00000000	0.00000000
3	8	3	0.00000000	0.00000000
4	8	3	0.00000000	0.00000000
1	9	1	-6.77844780	0.61305565
2	9	1	0.08370419	0.08299876
3	9	1	2.62574395	0.54775521
4	9	1	0.00000000	0.00000000
1	9	2	0.00000000	0.00000000
2	9	2	0.00000000	0.00000000
3	9	2	0.00000000	0.00000000
4	9	2	0.00000000	0.00000000
1	9	3	0.00000000	0.00000000

2	9	3	0.00000000	0.00000000
3	9	3	0.00000000	0.00000000
4	9	3	0.00000000	0.00000000
1	9	4	-6.49182586	0.79121319
2	9	4	0.25199287	0.10595408
3	9	4	3.68531114	0.72438949
4	9	4	0.00000000	0.00000000
1	9	5	-6.23467838	1.13137289
2	9	5	-0.09192577	0.15533805
3	9	5	5.93753802	1.06174572
4	9	5	0.00000000	0.00000000
1	10	1	0.36583629	0.32446125
2	10	1	0.38974703	0.05205723
3	10	1	-1.54744876	0.35967970
4	10	1	0.00000000	0.00000000
1	10	2	0.00000000	0.00000000
2	10	2	0.00000000	0.00000000
3	10	2	0.00000000	0.00000000
4	10	2	0.00000000	0.00000000
1	10	3	2.62157641	0.58380334
2	10	3	0.50152633	0.08979177
3	10	3	-3.07125173	0.62401819
4	10	3	0.00000000	0.00000000
1	11	1	0.00000000	0.00000000
2	11	1	0.00000000	0.00000000
3	11	1	0.00000000	0.00000000
4	11	1	1.69196396	0.17538689
1	11	2	0.00000000	0.00000000
2	11	2	0.00000000	0.00000000
3	11	2	0.00000000	0.00000000
4	11	2	1.87455382	0.17701458
1	11	3	0.00000000	0.00000000
2	11	3	0.00000000	0.00000000
3	11	3	0.00000000	0.00000000
4	11	3	1.86594409	0.17361762
1	11	4	0.00000000	0.00000000
2	11	4	0.00000000	0.00000000
3	11	4	0.00000000	0.00000000
4	11	4	1.66176943	0.17378112
1	11	5	0.00000000	0.00000000
2	11	5	0.00000000	0.00000000
3	11	5	0.00000000	0.00000000
4	11	5	1.82428459	0.17254502
1	11	6	0.00000000	0.00000000
2	11	6	0.00000000	0.00000000
3	11	6	0.00000000	0.00000000
4	11	6	1.14787107	0.17328923
1	12	1	0.00000000	0.00000000
2	12	1	0.00000000	0.00000000
3	12	1	0.00000000	0.00000000
4	12	1	1.23258839	0.05269246
1	12	2	0.00000000	0.00000000
2	12	2	0.00000000	0.00000000
3	12	2	0.00000000	0.00000000

4	12	2	0.27407409	0.05456789
1	12	3	0.00000000	0.00000000
2	12	3	0.00000000	0.00000000
3	12	3	0.00000000	0.00000000
4	12	3	1.49095715	0.05853813
1	12	4	0.00000000	0.00000000
2	12	4	0.00000000	0.00000000
3	12	4	0.00000000	0.00000000
4	12	4	1.15474690	0.04942064
1	12	5	0.00000000	0.00000000
2	12	5	0.00000000	0.00000000
3	12	5	0.00000000	0.00000000
4	12	5	0.00000000	0.00000000
1	12	6	0.00000000	0.00000000
2	12	6	0.00000000	0.00000000
3	12	6	0.00000000	0.00000000
4	12	6	0.00875980	0.05362063
1	12	7	0.00000000	0.00000000
2	12	7	0.00000000	0.00000000
3	12	7	0.00000000	0.00000000
4	12	7	1.37866025	0.07723315
